# Beneficial Effects of the Consumption of Hot-Water Extracts of Thinned Immature Mangos (*Mangifera indica* “Irwin”) on the Hypertriglyceridemia of Apolipoprotein E-Deficient Mice

**DOI:** 10.3390/metabo12020116

**Published:** 2022-01-25

**Authors:** Hayato Tajiri, Wataru Tanaka, Hiroki Matsuyama, Takuya Sugita, Kenta Hidaka, Daigo Yokoyama, Hiroyuki Sakakibara

**Affiliations:** 1 Graduate School of Agriculture, University of Miyazaki, 1-1 Gakuen-Kibanadai Nishi, Miyazaki 889-2192, Japan; gc16021@student.miyazaki-u.ac.jp (H.T.); gc14026@student.miyazaki-u.ac.jp (W.T.); nb21008@student.miyazaki-u.ac.jp (H.M.); yokoyama.d@cc.miyazaki-u.ac.jp (D.Y.); 2 Agricultural Administration Section, Saito 881-8501, Japan; sugitat@city.saito.lg.jp; 3 Star-Fruits Company, Ltd., Saito 881-0104, Japan; kentahidaka002@gmail.com

**Keywords:** apolipoprotein E-deficient mice, dyslipidemia, hypertriglyceridemia, thinned immature mango, triglyceride

## Abstract

The thinned immature fruit of the mango tree (*Mangifera indica* “Irwin”) are regarded as waste products. In this study, we evaluated the effects of daily consumption of a hot-water extract of thinned immature mango fruits (TIMEx) on the dyslipidemia of apolipoprotein E-deficient (ApoE^−/−^) mice. ApoE^−/−^ mice and wild-type BALB/c mice were fed a 20% fat diet containing 0%, 0.1%, or 1.0% TIMEx for 8 weeks. Their body mass, food intake, and water consumption were unaffected by the TIMEx. The 1.0% TIMEx supplementation significantly reduced serum triglyceride, but not total cholesterol concentration. This effect was significant in ApoE^−/−^ mice, but less marked under normal conditions in wild-type mice. In addition, the circulating concentrations of three hormones that regulate metabolism, resistin, leptin, and glucose-dependent insulinotropic polypeptide, were reduced by TIMEx consumption, which may be involved in its effect to prevent hypertriglyceridemia. However, none of the concentrations of TIMEx reduced the size of atherosclerotic plaque lesions. In conclusion, daily consumption of TIMEx ameliorates hypertriglyceridemia but not hypercholesterolemia in genetically predisposed mice.

## 1. Introduction

Dyslipidemia is characterized by a spectrum of quantitative and qualitative changes in lipids and lipoproteins [1]. The most common pattern of lipid abnormalities includes hypertriglyceridemia and hypercholesterolemia. Dyslipidemia is globally recognized as one of the most important risk factors for the development and complications of atherosclerotic cardiovascular disease, which is a major cause of morbidity and mortality [2,3,4]. One of the causes of dyslipidemia is recognized as a disturbed diet pattern. The diets of many populations around the world have moved toward a Western-style diet, which contains high proportions of fat and refined sugars, relatively low proportions of complex carbohydrates and fiber, and a low content of fruit and vegetables [5]. Therefore, modification of the diet is essential for the prevention of dyslipidemia.

Mango (*Mangifera indica* L.) is one of the most popular tropical fruits in the world. Global mango production was ~40 million tons in 2018, which represented an increase of 2.8% from 2017 [6]. The fruit pulp is characterized by density and sweetness, but also has high nutritional value, because of the presence of vitamins, dietary fiber, and various polyphenols [7,8,9,10]. Hence, the mango has been referred to as the “King of Fruits” [11].

For many fruits, including the mango, size is a major determinant of yield and marketability [12,13]. Most of the fruits are thinned out during their immature stage, which increases the size and quality of the remaining fruits [14]. In the mango production industry, the immature fruits, which are green and weigh <10 g, are harvested, and such fruits subsequently soften, but do not develop a pleasing flavor, and therefore cannot be sold [15]. Therefore, most thinned immature mango fruits are disposed of.

Our group has recently focused on the thinned immature fruits of the Irwin mango cultivar (*Mangifera indica* “Irwin”) as an unused natural resource. We conducted a 90-day toxicity study using rat model to evaluate the safety of a hot-water extract of thinned immature mango fruit (TIMEx), because there was insufficient published information concerning the safety and dietary characteristics of TIMEx to enable their dietary use. We found that 90 days of TIMEx administration was well tolerated by rats of both sexes and that it caused no significant changes in their condition, hematology, blood chemistry, or urine composition [16], and the No Observed Adverse Effect Level (NOAEL) for TIMEx in rats was calculated to be 2500 mg/kg/day. During the study, we also found that TIMEx reduced the serum triglyceride concentration; 90 days of administrating 2500 mg/kg/day TIMEx resulted in decreases in serum triglyceride concentration of 16.8% in male rats and 11.5% in female rats, by comparison with vehicle controls [16]. These results led us to formulate the hypothesis that TIMEx might prevent dyslipidemia, especially hypertriglyceridemia.

In this study, we evaluated the effects of the daily consumption of TIMEx on the dyslipidemia of atherosclerotic mice (apolipoprotein E-deficient mice; ApoE^−/−^ mice) that were consuming a 20% fat-containing diet. Additionally, we analyzed the secretion of major metabolic hormones including resistin, leptin, glucose-dependent insulinotropic polypeptide (GIP), insulin, amylin and glucagon-like peptide-1 (GLP-1), which regulate lipid homeostasis [17,18,19,20], in order to speculate the mechanisms involved.

## 2. Results

### 2.1. Growth Parameters and Organ Masses

The consumption of 0.1% and 1.0% TIMEx containing diet for 8 weeks did not affect the body mass gain, food intake, or water intake of either wild-type or ApoE^−/−^ mice (Table 1). The liver mass of wild-type mice that consumed 1.0% TIMEx diet increased, but the consumption of 0.1% TIMEx did not have this effect (Table 1). This effect did not occur in the ApoE^−/−^ mice. The masses of the kidney, spleen, visceral fat, and brown fat of both groups of mice were unaffected by TIMEx consumption.

### 2.2. Serum Lipid Concentrations

**Figure 1:**
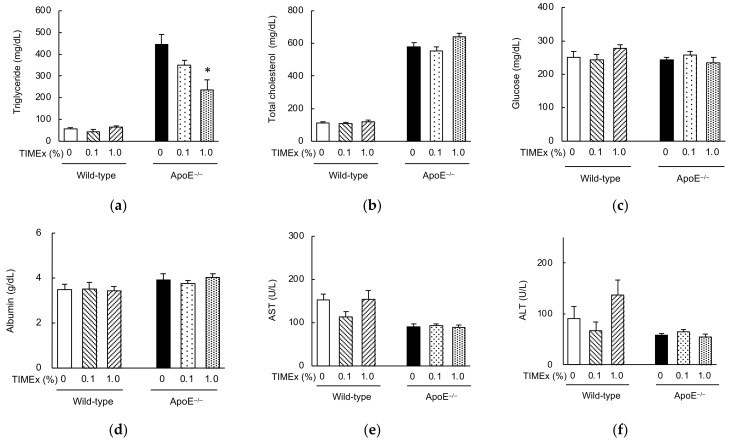
Effects of the consumption of a hot-water extract of thinned immature mangoes (TIMEx) on the serum biochemistry of apolipoprotein E-deficient (ApoE^−/−^) mice. ApoE^−/−^ mice and wild-type BALB/c mice were divided into three diet groups: an AIN-93M-based 20% fat diet containing 0%, 0.1%, or 1.0% TIMEx. A detailed description of the composition of each diet is shown in Table 2. Following 8-weeks consumption of their respective diets, mice were sacrificed without fasting. The data are mean ± SEM; *n* = 6 for wild-type mice and *n* = 9 for ApoE^−/−^ mice. (**a**) Triglycerides, (**b**) total cholesterol, (**c**) glucose, (**d**) albumin, (**e**) aspartate transaminase (AST), and (**f**) alanine transaminase (ALT). * *p* < 0.05 vs. the 0% TIMEx group; Williams’s multiple comparison test.

After 8 weeks consumption, the serum triglyceride and total cholesterol concentrations of the ApoE^−/−^ mice were much higher than those of wild-type mice fed the 0% TIMEx diet (446 ± 44 mg/dL vs. 57 ± 7 mg/dL for triglyceride; 578 ± 26 mg/dL vs. 114 ± 5 mg/dL for total cholesterol) (Figure 1a,b). The serum triglyceride concentration was dose-dependently reduced and was significantly affected in the 1.0% TIMEx group. However, this effect was not observed in the wild-type mice (57 ± 7 mg/dL for 0% TIMEx; 45 ± 10 mg/dL for 0.1% TIMEx; 65 ± 5 mg/dL for 1.0% TIMEx). On the other hand, TIMEx supplementation did not affect serum total cholesterol, glucose, albumin concentrations, nor the serum aspartate aminotransferase (AST), alanine aminotransferase (ALT) activities of the mice groups (Figure 1c–f).

### 2.3. Hepatic Lipid Concentrations

**Figure 2:**
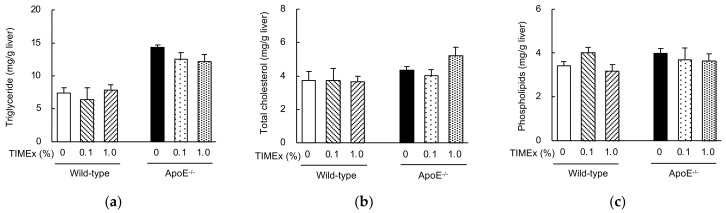
Effects of the consumption of a hot-water extract of thinned immature mangoes (TIMEx) on the hepatic lipid concentrations of apolipoprotein E-deficient (ApoE^−/−^) mice. ApoE^−/−^ mice and wild-type BALB/c mice were divided into three diet groups: an AIN-93M-based 20% fat diet containing 0%, 0.1%, or 1.0% TIMEx. A detailed description of the composition of each diet is shown in Table 2. Following 8-weeks consumption of their respective diets, mice were sacrificed without fasting. The data are mean ± SEM; *n* = 6 for wild-type mice and *n* = 9 for ApoE^−/−^ mice. (**a**) Triglyceride, (**b**) total cholesterol, and (**c**) phospholipid concentrations. There were no significant differences versus the control group (0% TIMEx).

The hepatic concentrations of triglycerides, total cholesterol, and phospholipids were measured. The hepatic triglyceride concentration was much higher in the ApoE^−/−^ mice than in wild-type mice fed the 0% TIMEx diet (14.3 ± 0.4 vs. 7.4 ± 0.8 mg/g) (Figure 2a). TIMEx supplementation tended to reduce triglyceride accumulation, but not significantly. There were no differences in the hepatic total cholesterol or phospholipid concentrations among the groups (Figure 2b,c).

### 2.4. Atherosclerotic Plaque Lesions

**Figure 3:**
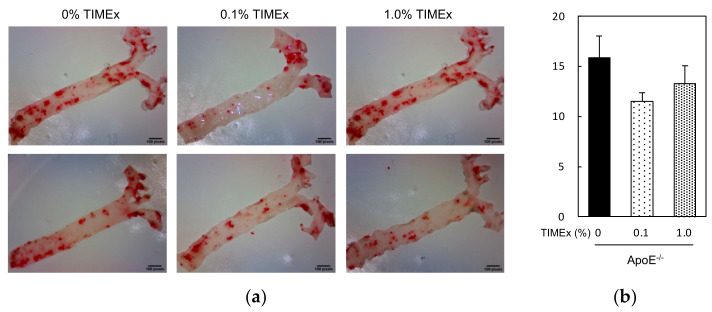
Effects of the consumption of a hot-water extract of thinned immature mangoes (TIMEx) on the atherosclerotic lesions of apolipoprotein E-deficient (ApoE^−/−^) mice. (**a**) Oil Red O-stained whole aortas and (**b**) the mean areas of the aortic atherosclerotic lesions, determined using ImageJ software (V. 1.44p, NIH, Bethesda, MD, USA). The data are mean ± SEM; *n* = 9. There were no significant differences vs. the control group (0% TIMEx).

We next analyzed the atherosclerotic plaque lesions in the aortas of the mice, measuring the percentage lipid deposition (Figure 3a). The atherosclerotic plaque lesions of the 0% TIMEx group (15.8 ± 2.2%) were similar to those of the 0.1% TIMEx group (11.5 ± 0.9%) and the 1.0% TIMEx group (13.3 ± 1.8%) after 8 weeks of consumption (Figure 3b).

### 2.5. Serum Hormone Concentrations

**Figure 4:**
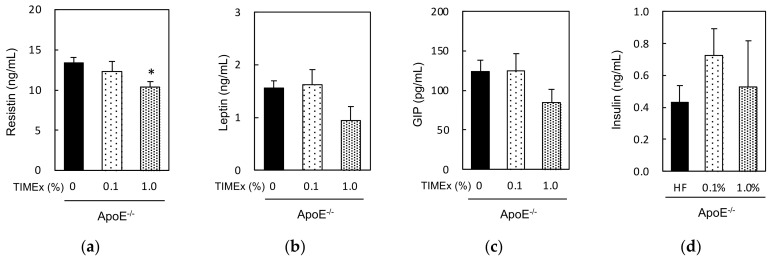
Effects of the consumption of a hot-water extract of thinned immature mangoes (TIMEx) on the serum concentrations of hormones in apolipoprotein E-deficient (ApoE^−/−^) mice. ApoE^−/−^ mice were divided into three diet groups: an AIN-93M-based 20% fat diet containing 0%, 0.1%, or 1.0% TIMEx. Following 8-weeks consumption, mice were sacrificed without fasting. The data are mean ± SEM; *n* = 9. (**a**) Resistin, (**b**) leptin, and (**c**) glucose-dependent insulinotropic polypeptide (GIP), (**d**) Insulin. * *p* < 0.05 vs. the control group (0% TIMEx); Williams’s multiple comparison test.

Finally, we measured the serum concentrations of resistin, leptin, GIP, insulin, amylin, and GLP-1 in ApoE^−/−^ mice that had consumed 0%, 0.1%, or 1.0% TIMEx-supplemented diets for 8 weeks (Figure 4). The resistin concentration was significantly lower in the 1.0% TIMEx group than in the 0%. The leptin and GIP concentrations also tended to be lower in the 1.0% TIMEx group, but not significantly. However, the consumption of 0.1% TIMEx did not have any effects on the concentrations of these hormones. Blood levels of insulin were not affected by consumption of TIMEx. Amylin and GLP-1 levels could not be fully detected in every sample (under the detection limits of 41 pg/mL) (data not shown).

## 3. Discussion

ApoE was initially described to be a lipid transport protein, and major ligand for low-density lipoprotein (LDL) receptors with a role in cholesterol metabolism and cardiovascular disease [21]. Hence, ApoE deficiency increases blood lipid concentrations [22]. However, the congenic ApoE deficient mice used in the present study are observed as being healthy and having a similar body mass comparing with their wild-type mice [23]. Our results are consistent with this background, because the body mass gain, daily food intake, and water consumption of the wild-type and ApoE^−/−^ mice were similar (Table 1). Nevertheless, the lipid profiles of the mice significantly differed: The serum triglyceride and total cholesterol concentrations of the ApoE^−/−^ mice were much higher than those of the wild-type mice (Figure 1a,b), as shown previously [24,25]. Thus, dyslipidemia developed specifically in ApoE^−/−^ mice, despite them consuming the same 20% fat containing diet as the wild-type mice.

Daily intake of a 1.0% TIMEx-supplemented diet increased the liver mass of the wild-type mice (Table 1), which might imply the development of hepatomegaly because of non-alcoholic fatty liver disease [26]. If this were true, hepatic fat accumulation would be associated with high circulating ALT and AST activities, which have been identified in individuals at risk of liver disease [27]. However, there were no differences in the activities in the wild-type mice consuming 0%, 0.1%, or 1.0% TIMEx-containing diets (Figure 1e,f). Therefore, we concluded that this effect was sporadic, but not toxic.

The daily consumption of TIMEx for 8 weeks significantly reduced the serum triglyceride concentration of ApoE^−/−^ mice, but not that of total cholesterol (Figure 1a). The effect on triglyceride was dose-dependent, with the effect of 1.0%-supplementation of the diet being significant. This effect was not the result of malnutrition, because there were no differences in food intake (Table 1) or serum albumin concentration (Figure 1d), which are used as markers of nutritional status, between the groups [28]. On the other hand, the hepatic triglyceride and total cholesterol levels were not affected by the consumption of TIMEx (Figure 2). The appropriate level of supplementation for animals cannot be extrapolated to determine a human equivalent dose on the basis of body mass alone. Instead, body surface area (BSA; mg/m^2^) is used to convert an animal dose to the equivalent human dose. The dose is multiplied by the Km values of 37 for adult humans and 3 for mice in order to convert a dose in mg/kg to a dose in mg/m^2^ [29], and calculated using the following formula:

(1) $$\mathrm{Human}\;\mathrm{equivalent}\;\mathrm{dose}\;(\mathrm{mg}/\mathrm{kg})\;=\;\mathrm{Mouse}\;\mathrm{dose}\;(\mathrm{mg}/\mathrm{kg})\;\times \;\frac{\mathrm{Mouse}\;\mathrm{Km}\;\left(3\right)}{\mathrm{Human}\;\mathrm{Km}\;\left(37\right)}$$

In the present study, the 1.0% TIMEx-supplemented group, in which final body mass was 28.1 ± 0.8 g, consumed 28.6 mg TIMEx per day (Table 1), equivalent to a daily consumption of TIMEx of 1000 mg/kg body mass in mice. This dose is equivalent to 81.1 mg TIMEx/kg body mass/day for humans, which equates to a 4860 mg dose for a 60 kg person. More detailed studies, particularly human trials, should be undertaken in the future.

TIMEx consumption did not affect the serum triglyceride concentration of wild-type mice. In a previous study, 90 days of TIMEx administration tended to reduce serum triglyceride in rats at a dose of 2500 mg/kg/day, although this effect was not significant [16]. In the present study, wild-type mice in the 1.0% group consumed daily 27.7 mg/mouse (955 mg/kg body mass) TIMEx (Table 1), indicating that the daily consumption of a larger amount of TIMEx might have a more marked effect on serum triglyceride in wild-type mice. Thus, the present data suggest that the daily consumption of 4860 mg TIMEx by adult humans would help prevent hypertriglyceridemia but would have a less marked effect under normal conditions. TIMEx used in this study was prepared by hot-water extraction of thinned immature mangos according to the green tea preparation method as described in the following Section 4.2.2. For human applications, we propose to consume the decoction of dried thinned immature mango powder. Fruit acidity of mango is attributed mainly to the concentration of citric acid [8]. Its amount is high in immature stage (ca. 1000 mg/100 fresh weight), and dramatically decrease during ripening process (ca. 100 mg/100 g fresh weight) [30]. Hence, the point to be paid attention is the strong and characteristic acidity.

An increase in LDL, which carries cholesterol in the circulation, provokes lipid accumulation in the vascular wall and plays a pivotal role in the progression of atherosclerosis [31]. Therefore, the daily consumption of food materials, such as walnuts, which reduce serum cholesterol and triglyceride concentrations, has been reported to reduce the atherosclerotic plaque area in ApoE^−/−^ mice [32]. However, even 1.0% TIMEx consumption for 8 weeks did not affect plaque area in the present study (Figure 3b). This implies that the daily consumption of TIMEx prevents hypertriglyceridemia, but not hypercholesterolemia, and therefore might not prevent atherosclerosis.

We also measured the concentrations of several hormones that regulate lipid homeostasis to better understand the effects of dietary TIMEx supplementation in ApoE^−/−^ mice. Resistin is produced by adipose tissue, and a high circulating concentration of resistin is associated with a higher risk of atherosclerosis [17,18]. Leptin is a hormone that is exclusively secreted by adipocytes, and its expression positively correlates with obesity [20]. GIP, which is secreted by enteroendocrine K-cells, has been reported to be implied in the development of obesity and the pathogenesis of cardiovascular disease [19]. Daily consumption of a 1.0% TIMEx-supplemented diet for 8 weeks significantly reduced the circulating resistin concentration in ApoE^−/−^ mice, and the concentrations of both leptin and GIP were also much lower (Figure 4a–c). The downregulation of these three hormones may be involved in the effect of TIMEx to prevent hypertriglyceridemia, but more detailed studies of the underlining mechanism of action should be undertaken in the future.

Recent studies have indicated that the gut microbes can impact host metabolism via signaling pathways in the gut, with effects on deposition of energy in fat stores, and consequently involves in both onset and progression of metabolic syndrome, including dyslipidemia and obesity [33,34,35]. Therefore, daily consumption of probiotics has been recognized to be a promising gut microbiota-targeted method for hyperlipidemia and atherosclerosis [36]. Some food materials and ingredients, such as *Luffa cylindrica* (L.) Roem, *Cordyceps militaris*, buckwheat and perilla oil, exert a potential dietary intervention strategy against obesity and dyslipidemia through modulating the gut microbiota [37,38,39,40]. Supplementation of mango pulp improves high-fat diet modulated gut microbiota [41]. Dietary fiber is major ingredient of mango (ca. 10 g/100 g of dry weight) with balance of soluble and insoluble [42]. Daily treatment of pectin, which is one of the major water-soluble dietary fiber in mango [8], exhibits improvements in intestinal microbiota and lipid metabolism in mice consumed high-fat diet [43]. Although information about bioactive compounds in thinned immature mangos have not been well elucidated yet, pectin levels are reported to be decreased during the ripening [11]. These observations indicate the possibility that one of major active ingredients for amelioration of hypertriglyceridemia might be pectin via regulation of gut microbiota. Additional candidate is mangiferin, which is rich-in mango pulp and reported to protect hyperlipidemia through the regulation of metabolic pathways including metabolism of dicarboxylate, reduction of apoptosis, and β-cell regeneration including glucose transporter 2 (Glut-2) [44,45].

In recent years, more attention has been paid to non-communicable diseases (NCDs), which cover many entities including cancers, type 2 diabetes and cardiovascular diseases, because of their high leading cause of death worldwide [46]. Widespread obesity and abdominal fat accumulation are a major public health problem because of its association with NCDs [47,48]. Our data also imply that daily consumption of TIMEx can impact the other NCDs via regulating of blood lipid levels.

## 4. Materials and Methods

### 4.1. Chemicals

Cellulose, α- and β-cornstarch, and sucrose were purchased from Oriental Yeast Co., Ltd. (Tokyo, Japan). t-Butylhydroquinone, casein, l-cystine, and soybean oil were from Wako Pure Chemical Industries, Ltd. (Osaka, Japan). The lard, mineral mixture (AIN-93M-Mix), and vitamin mixture (AIN-93-VX) were purchased from MP Biomedicals, LLC (Irvine, CA, USA). All other reagents were of the highest grade available.

### 4.2. Experimental Materials and Preparations

#### 4.2.1. Immature Mango Fruits

Immature Irwin mango fruits grown in Miyazaki, Japan, were collected by hand between the middle of February and the end of March 2018. After taxonomically identification on the basis of morphological characteristics by Mr. Kenta Hidaka (Star-Fruits Company, Ltd., Miyazaki, Japan), they were harvested under the following condition: the weight and size of individual fruits were <25 g and <Φ3 cm. The fresh samples were immediately transported to the laboratory, and hand-washed with tap water to remove dirt and dust. The cleaned whole fruits including peel, flesh and seed were rapidly frozen in liquid nitrogen, and then lyophilized using a freeze dryer (Dura-Top MP & Dura Dry MP, FTS SYSTEMS Ltd., Stone Ridge, NY, USA). Thinned immature mango were powdered using a Knife mill grindomix GM 200 (Verder Scientific Co., Tokyo, Japan), and stored away from light at 4 °C until extraction.

#### 4.2.2. Hot-Water Extraction

Extraction method was conducted according to the modified method for making the hot-water extract from green tea leaf [49]. In brief, thinned immature mango powder was mixed with ten volumes of hot water at 95 °C for 10 min with gently agitation. After centrifugation at 3000 rpm for 10 min, the supernatant was further filtrated with Whatman Grade GF/D Glass Microfiber Prefilters (Whatman, UK), and then lyophilized using the freeze dryer. The mango powder yield was ca. 26%. The lyophilized powder of hot-water extract from thinned immature mango (TIMEx) was stored at 4 °C until used in animal experiments.

### 4.3. Animal Experiments

#### 4.3.1. Institutional Approval of the Study Protocol

All animal procedures were approved by the Institutional Animal Care and Use Committee of the University of Miyazaki (No. 2018–027). This study was conducted in accordance with the Japanese Law for the Humane Treatment and Management of Animals (Law No. 105, 1973), which defines animal experimentation as the use of animals for scientific purposes and takes the 3Rs into consideration.

#### 4.3.2. Animal Housing, Diet, and Experiments

Male BALB/c. KOR/StmSlc-Apoeshl (ApoE^−/−^) mice and their corresponding wild-type male BALB/c. (ApoE^+/+^) mice (both 7 weeks old) were obtained from Japan SLC (Shizuoka, Japan). The animals were housed three per cage (width 235 mm × depth 165 mm × height 125 mm) in an air-conditioned animal housing room (temperature: 23 ± 2 °C; humidity: 55 ± 5%) under a 12-h dark/light cycle (light period: 9:00 AM to 9:00 PM), with free access to tap water and powdered AIN-93M diet (Table 2).

After 1-week acclimation, the ApoE^−/−^ and wild-type mice, respectively, were individually divided into two groups according to the modified methods reported by Hoang et al. [50]. The first group consumed a control diet (0% TIMEx), which was the modified AIN-93M based diet containing a 20% fat. The second groups consumed control diet containing 0.1% TIMEx or 1.0% TIMEx (Table 2). After 8-weeks consumption, blood sample was drawn from the abdominal vein under anesthesia with isoflurane (1.5%) without fasting and put into serum Capiject tubes (Terumo Medical Corporation, Somerset, NJ, USA). Following 30 min standing at room temperature, serum fraction was separated by centrifugation at 3500× *g* for 90 s and was stored at −80 °C until analysis. The organs including liver, kidney, spleen, and visceral fats (epididymal fat + perirenal fat) were weighed. Liver section was stored at −80 °C for lipid analysis after freezing in liquid nitrogen.

#### 4.3.3. Quantification of Atherosclerotic Lesions

Atherosclerotic lesion was assessed in the aortas using the modified methods of previous reports [51,52]. Briefly, after removing the organs listed above, mice were perfused with phosphate-buffered saline. The entire aorta was dissected from the proximal ascending aorta to the bifurcation of the iliac artery under a dissecting microscope (Stemi 305 cam, Carl Zeiss Microscopy GmbH, München, Germany). Adventitial fats were removed, and the aorta was opened longitudinally and pinned flat onto a silicon board. After fixing with 4% paraformaldehyde, the aortas were stained using Oil Red O. The staining aortas were photographed using a 1.2 mega pixel camera (Stemi 305 cam). The photographs were digitized, and total aortic areas and atherosclerotic lesion areas were calculated using Image J (V. 1.44p, NIH, Bethesda, MD, USA). The results were indicated as a percentage of the thoracic aortic area that contained lesions. This operation, including calculation were carried out by observers not informed about the kind of treatment each animal had received.

#### 4.3.4. Blood Chemistry and Adipokines

Serum biochemical parameters (triglycerides, total cholesterol, glucose, albumin, AST and ALT) were analyzed using a Dri-Chem 4000v chemistry analyzer (Fujifilm Co., Tokyo, Japan) with an individual cartridge slide. The serum resistin, leptin, glucose-dependent insulinotropic polypeptide (GIP), insulin, amylin and glucagon-like peptide-1 (GLP-1) levels were analyzed using an MMHMAG-44 Mouse Metabolic Hormone Panel multiplex biometric enzyme-linked immunosorbent assay (Millipore, Billerica, MA, USA) according to the manufacturer’s instructions. 

#### 4.3.5. Hepatic Lipid Analysis

Hepatic lipids were extracted according to our previous method [53]. Briefly, after 200 mg aliquots of liver samples were homogenized with 1 mL of 50 mM sodium acetate, 6 mL chloroform–methanol (2:1 [vol/vol]) was added, and the mixture incubated at 40 °C for 30 min. An aliquot of the organic phase (500 µL) was dried with a centrifugal concentrator (CC-105; Tomy Seiko Co., Ltd., Tokyo, Japan), and the residues were dissolved in 200 µL of 10% Triton X-100 containing isopropyl alcohol. Triglycerides, total cholesterol, and phospholipid levels were analyzed using individual test kits obtained from Wako Pure Chemical Industries, Ltd.

### 4.4. Statistical Analysis

Data are presented as the mean ± standard error of the mean (SEM). Statistical analyses were conducted using both software, Pharmaco ANOVA (ver. 1) (Scientist Press Co., Ltd., Tokyo, Japan) and Pharmaco Basic (ver. 16) (Scientist Press Co., Ltd.). Statistical significance against individual control diet (0% TIMEx) group was determined using one-way analysis of variance (ANOVA), followed by the Williams’ multiple comparison test as post-hoc test. To make comparisons within the groups, alpha value was set to 0.05.

## 5. Conclusions

In this study, we have determined whether TIMEx consumption protects against dyslipidemia, including hypertriglyceridemia and hypercholesterolemia, in ApoE^−/−^ mice. Our data show that the daily consumption of a 1.0% TIMEx-supplemented diet for 8 weeks prevents hypertriglyceridemia but not hypercholesterolemia in ApoE^−/−^ mice. However, this effect is less noticeable under normal conditions in wild-type mice. Lower concentrations of resistin, leptin, and GIP may mediate this effect. These findings imply that TIMEx is a novel candidate of functional foods. The next logical step should be undertaken in the future, particularly on the mechanism of action, as well as human trials.

## Figures and Tables

**Table 1:** Effects of the consumption of a hot-water extract of thinned immature mango on the growth parameters and organ masses of the mice.

Measurements	Wild-Type (*n* = 6)			ApoE^−/−^ (*n* = 9)		
	0% TIMEx	0.1% TIMEx	1.0% TIMEx	0% TIMEx	0.1% TIMEx	1.0% TIMEx
Body mass (g)						
week 0	23.9 ± 0.4	23.8 ± 0.4	23.9 ± 0.6	23.5 ± 0.5	23.2 ± 0.6	23.4 ± 0.3
week 8	29.3 ± 0.5	28.1 ± 0.7	29.0 ± 0.5	28.2 ± 2.3	28.4 ± 0.9	28.1 ± 0.8
Body mass gain (g)	5.4 ± 0.4	4.2 ± 0.5	5.1 ± 0.4	4.7 ± 0.7	5.3 ± 0.6	4.7 ± 0.6
Food intake (kcal/mouse/day)	12.1	12.0	13.7	12.9 ± 0.6	13.0 ± 0.3	14.2 ± 0.5
TIMEx consumption (mg/mouse/day)	-	2.6	27.7	-	2.8 ± 0.2	28.6 ± 1.0
Water intake (g/mouse/day)	2.9	2.4	2.7	2.7 ± 0.3	2.8 ± 0.3	3.3 ± 0.7
Relative organ masses (g/100 g body mass)						
liver	3.75 ± 0.09	3.58 ± 0.19	4.20 ± 0.08 *	4.88 ± 0.13	4.73 ± 0.10	4.31 ± 0.24
kidney	1.21 ± 0.222	1.40 ± 0.04	1.31 ± 0.04	1.43 ± 0.03	1.38 ± 0.08	1.41 ± 0.05
spleen	0.37 ± 0.07	0.46 ± 0.02	0.43 ± 0.02	0.46 ± 0.02	0.44 ± 0.01	0.41 ± 0.01
visceral fat	1.14 ± 0.23	1.02 ± 0.14	1.49 ± 0.16	2.08 ± 0.21	2.63 ± 0.36	2.22 ± 0.29
brown fat	0.26 ± 0.05	0.28 ± 0.01	0.35 ± 0.02	0.39 ± 0.04	0.39 ± 0.03	0.30 ± 0.05

TIMEx, hot-water extract from thinned immature mangoes. The data are mean ± SEM. * *p* < 0.05 vs. the 0% TIMEx group; Williams’s multiple comparison test.

**Table 2:** Composition of the AIN-93M–based experimental diets.

Ingredients	AIN-93M	Experimental Diets		
		0% TIMEx	0.1% TIMEx	1.0% TIMEx
β-Cornstarch (g)	46.6	30.6	30.5	29.6
α-Cornstarch (g)	15.5	15.5	15.5	15.5
Casein (g)	14.0	14.0	14.0	14.0
Soybean oil (g)	4.0	4.0	4.0	4.0
Lard (g)	-	16.0	16.0	16.0
Sucrose (g)	10.0	10.0	10.0	10.0
Cellulose (g)	5.0	5.0	5.0	5.0
Vitamin mixture (g)	1.0	1.0	1.0	1.0
Mineral mixture (g)	3.5	3.5	3.5	3.5
l-Cysteine (g)	0.18	0.18	0.18	0.18
Choline bitartrate (g)	0.25	0.25	0.25	0.25
t-Butylhydroquinone (g)	0.0008	0.0008	0.0008	0.0008
TIMEx (g)	-	-	0.1	1.0
Energy (kcal/g)	3.80	4.60	4.60	4.56

TIMEx, hot-water extract from thinning immature mango.

## Data Availability

No new data were created or analyzed in this study. Data sharing is not applicable to this article.
